# Biochemical Characteristics and a Genome-Scale Metabolic Model of an Indian Euryhaline Cyanobacterium with High Polyglucan Content

**DOI:** 10.3390/metabo10050177

**Published:** 2020-04-29

**Authors:** Ahmad Ahmad, Ruchi Pathania, Shireesh Srivastava

**Affiliations:** 1 DBT-ICGEB Center for Advanced Bioenergy Research, International Centre for Genetic Engineering and Biotechnology, New Delhi 110067, India; ahmadbioinfo@gmail.com; 2 Department of Biotechnology, Noida International University, Noida, U.P. 203201, India; 3 Systems Biology for Biofuels Group, International Centre for Genetic Engineering and Biotechnology, New Delhi 110067, India; ruchipathania5@gmail.com

**Keywords:** feedstock, biomass, marine, photosynthetic, polyglucans, fast growth

## Abstract

Marine cyanobacteria are promising microbes to capture and convert atmospheric CO_2_ and light into biomass and valuable industrial bio-products. Yet, reports on metabolic characteristics of non-model cyanobacteria are scarce. In this report, we show that an Indian euryhaline *Synechococcus* sp. BDU 130192 has biomass accumulation comparable to a model marine cyanobacterium and contains approximately double the amount of total carbohydrates, but significantly lower protein levels compared to *Synechococcus* sp. PCC 7002 cells. Based on its annotated chromosomal genome sequence, we present a genome scale metabolic model (GSMM) of this cyanobacterium, which we have named as *i*Syn706. The model includes 706 genes, 908 reactions, and 900 metabolites. The difference in the flux balance analysis (FBA) predicted flux distributions between *Synechococcus* sp. PCC 7002 and *Synechococcus* sp. BDU130192 strains mimicked the differences in their biomass compositions. Model-predicted oxygen evolution rate for *Synechococcus* sp. BDU130192 was found to be close to the experimentally-measured value. The model was analyzed to determine the potential of the strain for the production of various industrially-useful products without affecting growth significantly. This model will be helpful to researchers interested in understanding the metabolism as well as to design metabolic engineering strategies for the production of industrially-relevant compounds.

## 1. Introduction

Photosynthesis captures solar energy and converts atmospheric carbon dioxide into organic compounds [1]. Cyanobacteria are photosynthetic prokaryotes which may serve as biocatalysts for production of biofuels and biochemicals [2,3] due to their simple nutrient requirements [4], fast growth and easy transformability [5]. Marine cyanobacteria are more attractive as they do not compete with land and freshwater resources which can be limited in many countries [6]. Cyanobacterial carbohydrates can be used as a renewable feedstock [7,8,9] for fermentation to produce biochemicals and third/fourth generation biofuels. Glycogen, a polymer of glucose, is the primary storage compound in cyanobacteria [10]. Cyanobacterial biomass can be hydrolysed to yield glucose and amino acids, which can then be used for a variety of biotechnological purposes. For feedstock applications, one requires a cyanobacterium with fast growth rate and a higher polyglucan levels than reported yet. We have recently identified a marine cyanobacterium *Synechococcus* sp. BDU 130192 isolated from salt pans in India, which shows fast growth and a higher polyglucan content compared to *Synechococcus* sp. PCC 7002 (a fast-growing and model marine cyanobacterium) in normal photoautotrophic condition. Its genome has been sequenced [11].

The availability of its genome sequence makes it possible to identify the various genes present in the organism. A set of metabolic genes defines the metabolic capabilities of an organism. Genome-scale metabolic models (GSMMs) are large-scale stoichiometric models based on the annotated genome sequence that contain metabolic reactions present in the majority of pathways of the organism. These models allow qualitative predictions such as testing of gene essentiality and the metabolic responses to environmental perturbations, as well as quantitative predictions such as ratios of nutrient utilization, central carbon metabolism fluxes, cell growth and nutrient exchanges under different growth conditions [12]. Further, it is possible to incorporate kinetic data [13], gene expression and measured flux data [14,15] to these stoichiometric models in order to improve their quantitative predictive capacity. The estimation of intracellular fluxes or reaction rates is the first step towards quantitative understanding of metabolic response of the cell. The estimation of intracellular fluxes also provides useful insights for metabolic engineering and designing of heterologous pathways for enhanced production of biochemicals. Quantitative phenotype predictions from GSMMs have proven to be useful for bioengineering purposes. GSMMs, through the application of flux balance analysis (FBA) [16], have been widely used to estimate the intracellular fluxes and understand the metabolism of a number of organisms under different physiological and genetic conditions [17,18,19,20]. Due to their utility in analyzing the metabolic landscape of the organism of interest, GSMMs of various photosynthetic organisms, e.g., cyanobacteria (*Synechocyctis* [18,19], *Synechococcus* [21]), microalgae (*Chlamydomonas* [22,23], *Chlorella* [24,25] and *Nannochloropsis* [26,27]) have been reconstructed and used to analyze the process of photosynthesis and metabolism under a variety of conditions. The metabolic models of cyanobacteria have been exploited to understand their metabolism, and devise metabolic engineering strategies to synthesize and/or enhance the production of various platform biochemicals such as 1,3-propanediol [28], succinic acid [29] and ethanol [30]. The metabolic models of *Chlorella* [24,25], *Nannochloropsis* [26] and *Chlamydomonas* [22,23] have been used to explore their metabolism under different light and physiological conditions as they accumulate high amount of lipids [26]. Previous reconstructions of cyanobacterial GSMMs have utilized both a fully automated [31] as well as a manual curation [19]. However, as a previous report suggests, manual curation is required to produce good quality GSMMs even if most of the steps of reconstruction were conducted through automated tools [32].

In this work, we compare the characteristics such as growth, biomass composition, cell-surface structure, oxygen evolution rates and glycogen productivity of the Indian marine cyanobacterium *Synechococcus* sp. BDU 130192 to that of *Synechococcus* sp. PCC 7002, a fast-growing model marine cyanobacterium. We also present a manually-curated genome-scale metabolic model of *Synechococcus* sp. BDU 130192, *i*Syn706. The GSMM was used to compare the intracellular flux distributions of the two marine cyanobacteria. Lastly, the model was employed to determine the biotechnological potential of the native cyanobacterium for various bioproducts and to determine engineering targets for the production of heterologous compounds.

## 2. Results

### 2.1. Growth and Total Carbohydrate Content of Synechococcus sp. BDU 130192 and Synechococcus sp. PCC 7002

**Figure 1:**
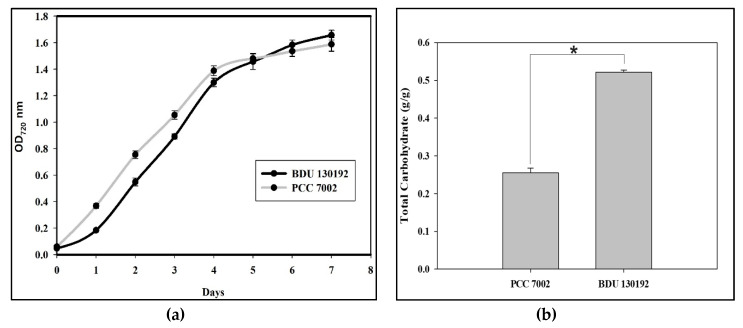
Comparison of two *Synechococcus* strains, BDU 130192 and PCC 7002 with air bubbling: (**a**) growth pattern; (**b**) Total carbohydrate measured when OD_720_ reached to 1. Data presented as mean ± s.d. of three independent replicates. (* shows *p* < 0.005 value).

Under the optimal conditions for growth (as defined in the methods section) with air bubbling, *Synechococcus* sp. BDU 130192 exhibits biomass accumulation similar to that of *Synechococcus* sp. PCC 7002 (Figure 1a), while the total carbohydrate (g/g) was about two fold higher (Figure 1b).

### 2.2. Structural analysis of Synechococcus sp. BDU 130192 and PCC 7002

**Figure 2:**
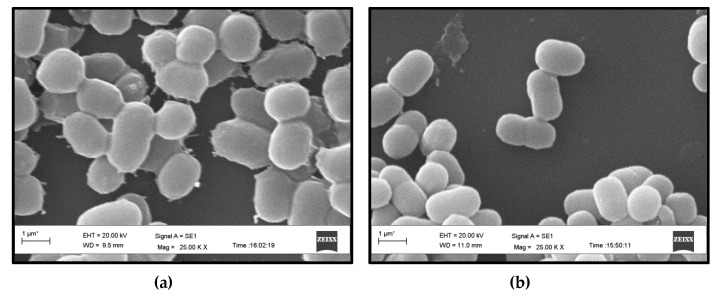
Scanning electron microscope analysis of: (**a**) *Synechococcus* sp. BDU 130192; (**b**) *Synechococcus* sp. PCC 7002.

*Synechococcus* sp. BDU 130192 cells have a comparable size to that of *Synechococcus* sp. PCC 7002 cells (Figure 2). However, the surface of *Synechococcus* sp. BDU 130192 cells seemed rougher and the cell wall seemed to contain larger amounts of exopolysaccharides (EPS) compared to the *Synechococcus* sp. PCC 7002 cells, though this was not quantified.

### 2.3. Oxygen Evolution Rate and Dark Respiration Rate

Measuring the rate of oxygen evolution provides an indication of how well the photosystem II (PSII) is functioning and at what rate the electrons are being produced at PSII at a particular light intensity. We compared the oxygen-evolving activities of both the strains. The rate of photosynthetic oxygen evolution for *Synechococcus* sp. BDU 130192 was 1.65 ± 0.07 mmol∙(gDCW∙h)^−1^ at 34 °C and for *Synechococcus* sp. PCC 7002 was 1.89 ± 0.06 mmol∙(gDCW∙h)^−1^ at 38 °C (*p* < 0.05, *t*-test). There was a remarkable difference in the respiration activity, which was estimated for *Synechococcus* sp. BDU 130192 as 1.38 ± 0.03 mmol∙(gDCW∙h)^−1^ and for *Synechococcus* sp. PCC 7002 as 0.868 ± 0.05 mmol∙(gDCW∙h)^−1^ (*p* < 0.05, *t*-test).

### 2.4. Glycogen Synthesis Genes Transcript Levels

**Figure 3:**
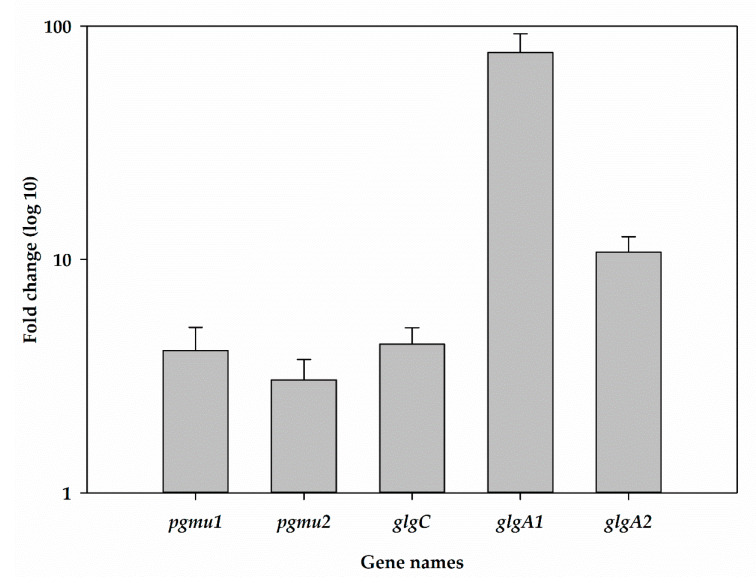
Relative levels of transcripts of genes involved in glycogen synthesis in *Synechococcus* sp. BDU 130192 compared to *Synechococcus* sp. PCC 7002.

The synthesis of glycogen from glucose-6-phosphate is catalysed by three enzymes: phosphoglucomutase (PGMU) that conducts the isomerization of G-6-P to G-1-P, glgC catalyses the ADP-glucose pyrophosphorylase, the enzyme that converts G-1-P to ADP-Glucose, and glgA encodes for glycogen synthase. The analysis of transcripts levels of glycogen-synthesis genes of *Synechococcus* sp. BDU 130192 and *Synechococcus* sp. PCC 7002 showed that the levels of *PGMU 1* is approximately 4 fold higher, *PGMU 2* is 3-fold, *glgC* is 4-fold, *glgA1* is 77-fold and *glgA2* is 10-fold higher in *Synechococcus* sp. BDU 130192 vs. in *Synechococcus* sp. PCC 7002 cells (Figure 3). Thus, the higher polyglucan levels were associated with a higher expression of glycogen-synthesis genes.

### 2.5. Biomass Composition of Synechococcus sp. BDU 130192 and Its Comparison to That of Synechococcus sp. PCC 7002

The dry cell weight of *Synechococcus* sp. BDU 130192 was 0.265 ± 0.002 g/l/OD while that of *Synechococcus* sp. PCC 7002 was 0.318 ± 0.022 g/l/OD (*p* < 0.05, *t*-test). The measurement of biomass composition of *Synechococcus* sp. PCC 7002 and *Synechococcus* sp. BDU 130192 showed that *Synechococcus* sp. BDU 130192 has significantly high levels of polyglucans and DNA but reduced levels of other biomass components compared to *Synechococcus* sp. PCC 7002 (Table 1). The presence of approximately two-fold levels of DNA in *Synechococcus* sp. BDU 130192 compared to *Synechococcus* sp. PCC 7002 could be due to diploidy in *Synechococcus* sp. BDU 130192 as seen in some other cyanobacteria [33] but remains to be measured. The biomass formula and the degree of reduction for the *Synechococcus* sp. BDU 130192 were calculated to be CH_1.59_O_0.57_N_0.13_P_0.004_S_0.002_ and 4.11, respectively. Our measured biomass composition for *Synechococcus* sp. PCC 7002 cells is fairly comparable to that reported by [34,35] though the total carbohydrates estimated by us are higher in *Synechococcus* sp. PCC 7002 and chlorophyll *a* is slightly lower. However, the measured composition is quite different from that reported by Beck et al. [36]. Culture conditions and measurement methods can affect the measured biomass composition of cyanobacteria [35] and could be the reasons in the observed differences in biomass compositions across various studies for *Synechococcus* sp. PCC 7002.

### 2.6. Phylogenetic Analysis of Synechococcus sp. BDU 130192

**Figure 4:**
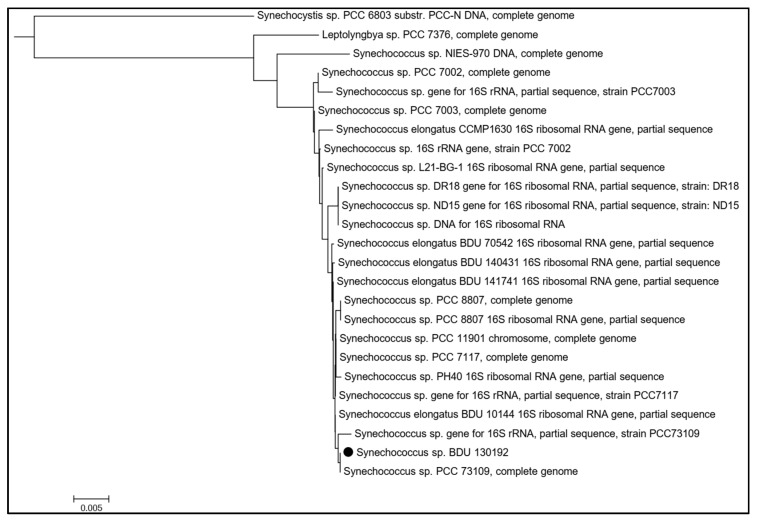
Phylogenetic positioning of *Synechococcus* sp. BDU 130192 based on NCBI database.

The phylogenetic tree obtained by the BLAST search revealed that the strain *Synechococcus sp.* BDU 130192 was a close relative of *Synechococcus sp.* PCC 73109 (Figure 4).

### 2.7. Gap-Filling and General Properties of the Model

#### 2.7.1. Analysis of Gap-Filling Reactions

There were 61 enzymes which were required for biomass-precursor biosynthesis but were absent in the genome annotation. BLAST searches against *Synechococcus* sp. PCC 7002 or *Synechocystis* sp. PCC 6803 genes supported the annotation of 32 of these enzymes while the remaining 29 enzymes could not be annotated. The 29 gap-filling enzymes were associated with 56 gap-filling reactions, which represent <7% of the total 908 reactions. In addition, one non-enzymatic and one each of demand and sink reactions are also present in the model. Thus, the model has 59 reactions without genetic evidence. Out of these 59 reactions, 31 reactions are orphan ones, even in the *Synechococcus* sp. PCC 7002 and *Synechocystis* sp. PCC 6803 GSMMs. A list of all the gap-filling reactions is provided in the Supplementary Materials File S4.

#### 2.7.2. General Properties of the Model

**Figure 5:**
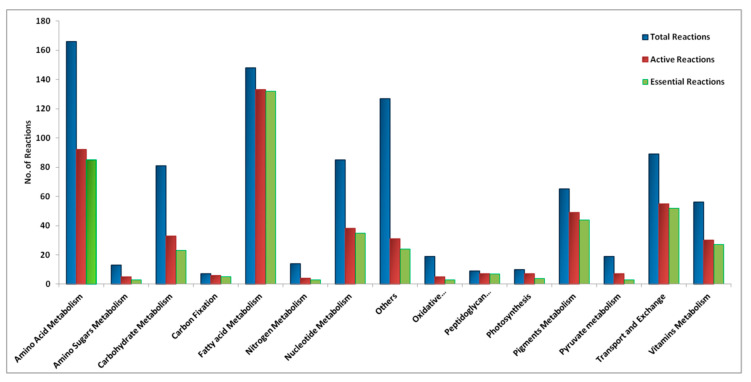
Distribution of total, active and essential reactions across various subsystems in *i*Syn706.

The reconstructed model, *i*Syn706, contains 706 genes, 908 reactions and 900 metabolites. The model comprises of 819 metabolic, 66 transport and 23 exchange reactions. Table 2 compares the size of our model with those of other published cyanobacterial GSMMs. The Supplementary Files S1, S2 and S3 contain the model as an SBML, Excel and the Matlab-readable mat formats, respectively. A detailed pathway-wise comparison of our model with *i*Syp708 model is provided in the Supplementary File S7. Our model generally has a greater number of reactions in the pathways for amino acid metabolism, carbohydrate metabolism, folate and riboflavin metabolism. The model is compatible with several modelling packages, e.g., COBRA Toolbox [37], ScrumPy [38], and Sybil [39]. The model has four compartments, namely extracellular, cytosol, periplasm and thylakoid. Most reactions (797) operate in cytosol while 15 reactions involving the photosynthesis and respiration are located in thylakoid, the 73 transport reactions are located in periplasm. The metabolic reactions present in the model have been categorised into 15 different subsystems (Figure 5). Out of the total 15 subsystems, the amino acid metabolism pathway has the highest number of reactions (165) followed by the fatty acid metabolism pathway (148).

### 2.8. Model Simulations

For photoautotrophic simulations, the growth rate was fixed at the measured value of 0.051 h^−1^, while the O_2_ evolution rate was allowed to vary between 1.6 ± 1 mmol∙(gDCW∙h)^−1^, based on the experimentally measured value. This led to a calculated CO_2_ uptake rate of 1.92 mmol∙(gDCW∙h)^−1^, photon uptake rate of 25.45 mmol∙(gDCW∙h)^−1^, and nitrate uptake rate of 0.22 mmol∙(gDCW∙h)^−1^. The model simulations predicted an O_2_ release flux of 2.43 mmol∙(gDCW∙h)^−1^_._ Thus, the O_2_/CO_2_ ratio was predicted to be 1.26 compared to 1.05 with the *i*Syp708 model and 1.5 in the *i*JN678 model.

The model simulation revealed that overall, 502 reactions are active under photoautotrophic condition. 133 out of the 148 reactions of fatty acid metabolism are active, making it the most active subsystem followed by the subsystem of amino acid metabolism with 92 out of the total 165 reactions as shown in Figure 5.

### 2.9. Reaction Deletion Analysis to Identify Essential Reactions

A total of 450 reactions were predicted as essential for growth in photoautotrophic condition. A further analysis of these essential reactions revealed that the fatty acid metabolism pathway has the highest numbers of essential reactions followed by the amino acid metabolism pathway. Figure 5 shows the distributions of total, active and essential reactions under photoautotrophic condition across different pathways.

We also performed the reaction essentiality analysis for the *i*Syp708 model and found that 83 essential reactions involved in amino acid metabolism, 19 involved in carbohydrate metabolism, 52 in fatty acid metabolism, 36 in nucleotide metabolism and 10 reactions involved in vitamins metabolism were essential in that model. Overall, there are 277 out of 648 reactions (~42%) that are essential in the *i*Syp708 model against 450 reactions (~50%) in the *i*Syn706 model. We investigated the reason for less essential reactions in *i*Syp708 model and found that the model has lumped reactions while *i*Syn706 has reactions for each step of fatty acid synthesis, i.e., initiation, elongation and termination. Thus, all the reactions of these three steps are essential due to their roles in biomass precursor biosynthesis in *i*Syn706 model while fewer reactions (lumped) are essential in *i*Syp708 model. However, the fraction of fatty acid and pigment metabolism reactions that were essential in our model was similar to that in *i*Syp708 (for *i*Syn706: 0.8 and for *i*Syp708: 0.89 of the total reactions present in this subsystem). Keeping the expanded form of fatty acid metabolism reactions in the model makes it more accurate and makes it possible to study the effect of different model parameters (e.g., growth rate, light intensity etc.) on the fatty acid metabolism reactions.

### 2.10. Detailing Metabolism under Photoautotrophic Condition and the Maximum Theoretical Yields of Native and Heterologous Compounds

**Figure 6:**
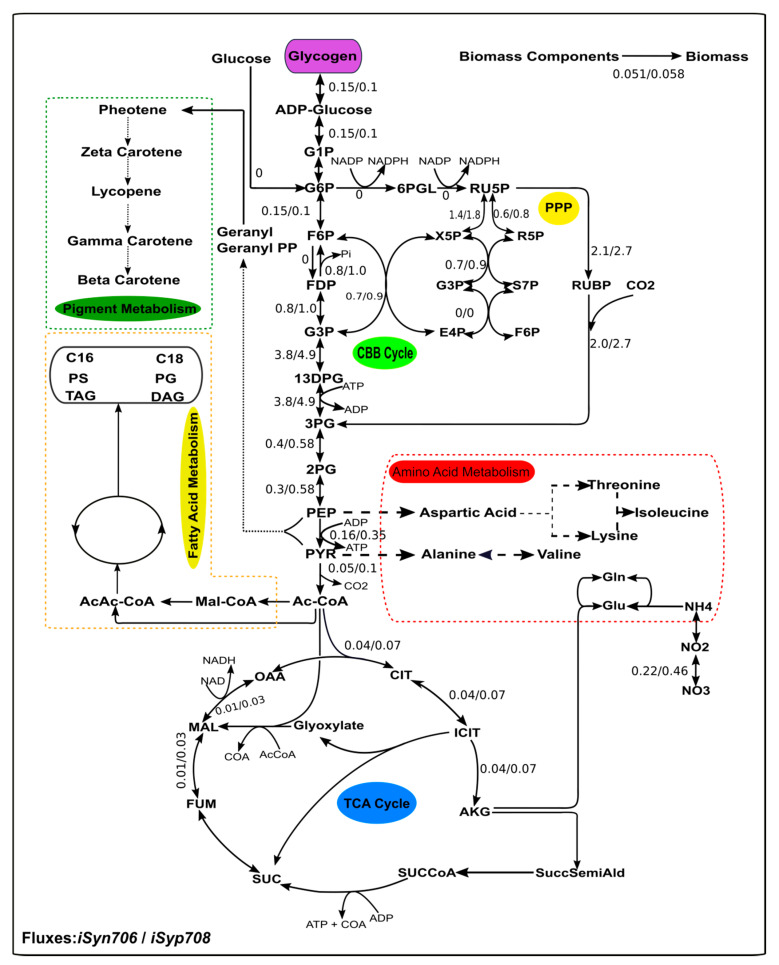
Simulated Flux map under photoautotrophic condition for *i*Syn706 and *i*Syp708 models. The growth rates of both the models were fixed to the experimentally determined values of 0.051 h^−1^ and 0.058 h^−1^ for *i*Syn706 and *i*Syp708, respectively while minimisation of the total fluxes was chosen as the objective function during simulations.

A detailed analysis of the model simulated under photoautotrophic condition showed CO_2_ fixation by C3 cycle, utilising ATP and NADPH produced during the light reactions. The Calvin Benson Bassham (CBB) cycle is almost equally active in both the models. We compared the flux distributions of the central Carbon metabolism reactions in both *i*Syp708 and *i*Syn706 models (Figure 6) by minimizing the total flux required to mimic the measured growth rates. The differences in the calculated flux distributions reflected the differences in the measured biomass compositions. The flux through glycogen synthesis pathway in *i*Syn706 was simulated to be almost double to that in the *i*Syp708 model, while the fluxes through the tricarboxylic acid cycle (TCA) are slightly more in *i*Syp708 in comparison to *i*Syn706 model. Similarly, the NO_3_^−^ intake in *i*Syn706 was about half of that in *i*Syp708, reflecting the reduced protein content in the former.

A unique feature of cyanobacteria is their unusual TCA cycle [42]. Instead of the alpha ketoglutarate dehydrogenase enzyme, cyanobacteria have 2-oxoglutarate decarboxylase (2OGDC) and succinyl semialdehyde dehydrogenase (SSADH) enzymes which shunt carbon from 2-oxoglutarate to succinate via succinyl semialdehyde (SSA). Interestingly, the simulation results show that only a part of the TCA cycle is active in *i*Syn706 in spite of the presence of the 2OGDC and SSADH shunt in simulated photoautotrophic conditions. This result is in agreement with a previous study [34] which showed a very low flux (0–0.1 mmol∙(gDCW∙h)^−1^) through both of these reactions under photoautotrophic conditions. A fraction of the total uptake flux goes into the TCA cycle via AcCoA. The flux from 2-OG (alpha-KG) is diverted to the GS-GOGAT (glutamine synthase-2-oxoglutarate amido transferase) cycle. The bacterium assimilates NO_3_^−^, converts it into NH_4_^+^ and finally into glutamine and glutamate via the GS-GOGAT cycle by the action of the enzymes glutamine synthase and glutamate 2-oxoglutarate amido transferase, respectively.

The synthesis of fatty acids is initiated by Acetyl-CoA, 3% of the total carbon intake goes to fatty acid metabolism and produces different types of fatty acids. The low value of flux through the fatty acid metabolism is in line with the low lipid levels seen in this organism. PEP and pyruvate form farnesyl pyrophosphate (FPP) through a cascade of reactions which in turn gets converted through a series of reactions into carotenoids like beta-carotenoids. A very small amount of flux goes to pigments metabolism. Cyanobacteria are promising organisms to work as photosynthetic cellular factories [43,44]. They can potentially synthesise diverse native and non-native industrially-relevant products (e.g., solvents, biofuels and food additives etc., Table S2) [43,44]. Flux balance analysis (FBA) was applied on the model *i*Syn706 to predict the theoretical yields (mol product produced/mol CO_2_ consumed) for four native and six non- native products under phototrophic condition (Supplementary File S7). The growth rates were fixed at 80% of the wild type growth rate. The yields of the compounds tested were inversely proportional to the number of C in the molecule. Acetate was the native compound produced at the highest yield while ethanol was the heterologous compound produced at the highest yield. These analyses identified the metabolic capabilities of the organism and the minimum number of gene additions needed to produce heterologous compounds.

## 3. Discussion

Cyanobacteria are potential feedstocks for biotechnological processes. However, there is a need to investigate, identify and develop non-model strains that show unique characteristics. In this study, we report a strain isolated from Indian salt pans that shows growth comparable to *Synechococcus* sp. PCC 7002, but has higher polyglucan content under normal growth conditions. Identifying such a potential organism is a first step in subsequent process and genetic engineering strategies to improve the growth rates of photoautotrophic microbes. We have measured the macromolecular composition of biomass of this organism and also created a GSMM for this cyanobacterium to help in understanding its metabolic capabilities.

Generally, in bacteria, including many cyanobacteria, proteins are the biomass components present in the greatest amounts. Upon nitrogen starvation, there is a breakdown of proteins and the carbon is stored in the form of polyglucans. Interestingly, the glycogen and total carbohydrates observed in *Synechococcus* sp. BDU 130192 under normal growth conditions are comparable to those observed in other strains after nitrogen/phosphorus deprivation. The elevated polyglucan level is associated with a lesser degree of reduction compared to that reported for *Synechococcus* sp. PCC 7002 [45]. In agreement with the elevated levels of carbohydrates and glycogen, the expression of the glycogen-synthesizing genes is higher. *Synechococcus* sp. BDU 130192 contains significantly lower amounts of proteins compared to *Synechococcus* sp. PCC 7002 while the growth rate is comparable. Whether the faster growth is because of efficient use of the proteome will require further studies. Similarly, currently it is not clear why *Synechococcus* sp. BDU 130192 cells showed comparable growth to *Synechococcus* sp. PCC 7002 cells even though the chlorophyll *a* levels in the former are about a third of those in the latter. It appears that the cells exhibit a nutrient-limiting behaviour (reduced protein and chlorophyll, elevated storage polymers) much earlier compared to *Synechococcus* sp. PCC 7002 cells and while still in growth phase.

The strain was identified to be closely related to *Synechococcus* sp. PCC 73109, a strain that, unlike *Synechococcus* sp. PCC 7002, is not auxotrophic to vitamin B_12_. It has been shown in *Synechococcus* sp. PCC 7002 that vitamin B_12_ is primarily used as a cofactor in methionine biosynthesis [46]. The methionine synthase has two isoforms: metH (E.C. 2.1.1.13) that is cobalamin dependent, and metE (E.C. 2.1.1.14) that is cobalamin-independent. The *Synechococcus* sp. BDU 130192 genome has two isoforms of metE (reactions R04405 and R09365 in the model) and hence should not require vitamin B_12_ for growth. Our preliminary experiments indicate that *Synechococcus* sp. BDU 130192 grows well in medium lacking vitamin B_12_ (results not shown), which will result in reduced cost of medium for this organism.

The strain was isolated from salt pans. There are several indicators that suggest that *Synechococcus* sp. BDU 130192 will have good osmotic and ionic stress tolerance. This includes higher carotenoids levels, a rougher surface suggestive of a thicker membrane and elevated levels of polyglucans. Genome-wise, 5% of the detected genes belong to the “stress metabolism” subsystem. It will be of interest to investigate the salt tolerance levels of this organism because an ability to grow at high salt concentrations may be advantageous when growing cultures in non-axenic conditions such as in open ponds. This stress-tolerance comes at a cost of higher maintenance ATP as suggested by the higher dark respiration rates of this strain. The elevated glycogen levels may be there to supply the excess ATP required during dark phase. However, for biotechnological applications where the cells may be grown in photo bioreactors under constant illumination, the excess glycogen stored would lead to greater glycogen productivity, as shown in this study.

The detailed, manually-curated genome-scale metabolic model will also help in understanding the metabolic capabilities for photoautotrophic production of biochemicals. Currently, the model employs GAM and NGAM values based on *Synechococcus* sp. PCC 7002, which may be oversimplification, but a common practice for cyanobacterial models [35,40]. It is likely that the GAM of *Synechococcus* sp. BDU 130192 is lower than that of *Synechococcus* sp. PCC 7002 (due to its lower protein levels) and the NGAM is higher (as suggested by a higher oxygen consumption in dark). We investigated the effect of lower GAM (35 and 40 mmol∙(gDCW)^−1^) and higher NGAM (3, 4, 5 mmol∙(gDCW∙h)^−1^). Our results suggest that mainly the photon intake and oxygen evolution change slightly, while the underlying flux distribution, which is a function of biomass equation and metabolic network, isn’t altered significantly. Yet, the inclusion of precise GAM and NGAM values for the particular strain will make the GSMM even more accurate. Currently, the oxygen evolution predicted by the model is different from the measured value with the model over-predicting the O_2_ production. The reason for this divergence is not clear, but may involve some errors in the biomass composition measurement [45]. The oxygen evolution based on the calculation of photosynthesis quotient, i.e., the ratio of O_2_ produced to CO_2_ intake, [47] utilizing the degree of reduction was 2.06 mmol∙(gDCW∙h)^−1^, a value in-between the measured one and that predicted by the GSMM. Therefore, an error in the measurement of O_2_ evolution rate could not be entirely ruled out.

Another area for improvement is further characterization and gene-assignment for the orphan reactions. Not surprisingly, some important central metabolic reactions are orphan in this model, as well as in other models. This includes reactions in the lipid and amino acid metabolism, as well as 2-oxoglutarate decarboxylase (2-OGDC, E.C. 4.1.1.71). In *Synechococcus* sp. PCC 7002, earlier the gene locus SYNPCC7002_A2770 was shown to code for acetolactate synthase (E.C. 2.2.1.6) as well as 2-OGDC [42]. Similarly, the locus SYNPCC7002_A1531 corresponds to 2-succinyl-6- hydroxy-2,4-cyclohexadiene-1-carboxylic acid synthase/2-oxoglutarate decarboxylase (accession No. CP000951). We could find the sequences homologous to both these sequences in the *Synechococcus* sp. BDU 130192 genome. These results strongly suggest the presence of the 2-OGDC reaction in the metabolic network of *Synechococcus* sp. BDU 130192. However, because an exact locus cannot be assigned to the 2-OGDC gene yet, this reaction is designated as an orphan reaction. Under conditions of localized high O_2_ concentration, RuBisCO can catalyse the photorespiration reaction, i.e., oxidation of 3-PGA to generate 2-phosphoglycolate (2-PG). As such, a very small flux was predicted for the oxygenase reaction of RuBisCO. The 2-PG is further metabolized to glycolate via phosphoglycolate phosphatase (PGP) reaction present in the model. Cyanobacteria have three pathways for metabolism of glycolate, viz. the canonical C2 cycle that is common with algae and higher plants, the decarboxylation pathway and the glycerate pathway [36]. However, none of these pathways are complete in the model because homologs of some of the enzymes could not be identified in the genome. For example, homologs of the following enzymes were not found: hydroxypyruvate reductase (HPR1) in the C2 cycle, glyoxylate carboxyligase in the glycerate pathway, and oxalate decarboxylase and formate reductase of the decarboxylation pathway. All the other reactions of the C2 cycle were present and the tartronate semialdehyde reductase (TSR) reaction of the glycerate pathway was present. Beck et al. [36] have shown that HPR1 is expressed in *Thermosynechococcus* sp. though it is not clear whether HPR1 is expressed in *Synechococcus* sp. BDU 130192. Addition of the HPR1 reaction to the model made no difference to the flux distribution (not shown). Nonetheless, similar additions/ deletions could be incorporated in the model and tested as further evidence of their existence in this specie becomes available.

The model was analysed through FBA, which simulates the flux distribution under conditions of some assumed optimality (e.g., minimization of total flux as in this case). The availability of a GSMM makes it possible to conduct other metabolic network analyses such as elementary flux mode (EFM) analysis which can comprehensively cover all possible metabolic states of a network.

We also performed reaction essentiality analysis and interestingly, we identified that close to half of the total reactions in the model were essential. The number of essential reactions within each metabolic pathway agrees well with the number of reactions carrying flux (active reactions). The large fraction of essential reactions shows the relative non-redundancy of the metabolic network, typically associated with lower organisms. The number of reactions in the subsystem of fatty acid metabolism is higher in our model as every fatty acid synthesis reaction is included, unlike in some other models [35,40] where the fatty acid synthesis is lumped together. This leads to a greater number of fatty acid synthesis reactions being essential in our model. However, the fraction of essential reactions to the total number of active reactions is comparable (0.90 of the total reactions present in this subsystem in ours vs. 0.86 in *i*Syp708).

We have identified the potential yields of some compounds using the reconstructed GSMM. The yields are similar to that got with *i*Syp708. Manual identification of the genes to be added was done here due to a limited number of compounds tested. However, computational strain design methods are available [48] and the model can be utilized in those too. Additionally, in *Synechococcus* sp. BDU 130192, the flux going towards glycogen/ polyglucans can be easily channelled away from the storage compounds towards target product, increasing the potential yields. This would be especially useful when cells are grown in continuous illumination. Systems for genetic engineering of *Synechococcus* species are available. It is expected that the systems could be applied or adapted for this strain too, though this needs to be tested.

Overall, this work presents the general properties of the biomass composition of a non-model *Synechococcus* species and provides its GSMM. The organism shows potential for development as a feedstock organism while the availability of its GSMM will help in systems-level analysis and metabolic engineering of this strain.

## 4. Materials and Methods

### 4.1. Culture Conditions

*Synechococcus* sp. BDU 130192 was obtained from the National Facility for Marine Cyanobacteria (NFMC) at Bharathidasan University (Tiruchirapally, India) and the *Synechococcus* sp. PCC 7002 was obtained from the Pasteur Culture Collection (PCC, Paris, France). The strains were inoculated in A^+^ medium (supplemented with Vit. B_12_, initial pH = 8.2) at 0.05 OD_720_ nm and grown in a Multi-cultivator (MC 1000-OD, PSI instruments, Drasov, Czech Republic). *Synechococcus* sp. BDU 130192 was cultured at 34 °C and continuously illuminated with cool white LED lights at 300 μmol∙m^−2^∙s^−1^, while the *Synechococcus* sp. PCC 7002 cells were cultured at 38 °C under 250 μmol∙m^−2^∙s^−1^ of illumination. The cultures were aerated at a rate of 0.5 mL per min using compressed air. Cell growth was observed at OD_720_ nm after every 24 h for 7 days.

### 4.2. Microscopic Analysis of Cyanobacterial Cells Using Scanning Electron Microscopy (SEM)

The exponentially growing cells were harvested at 7000 *g* and washed with 0.1 M sodium phosphate buffer (pH = 7.2) and fixed using in 3% glutaraldehyde for 2 h at 4 °C. The cells were then treated with 1% osmium tetraoxide, dehydrated in ethanol, dried with an air dryer, mounted on a specimen stub, coated with sputter coater gold coating unit (POLARON SC7640, Quorum Technologies, Newhaven, East Sussex, UK) and imaged under SEM (Carl Zeiss EVO 40 used at 20 kV, Jena Germany).

### 4.3. Measurement of Oxygen Evolution and Dark Respiration Rates

Photosynthetic oxygen evolution and respiration rates were measured by using a dissolved oxygen (DO) probe (Applikon Biotechnology, Delft, The Netherlands). The cells were grown to mid exponential phase, harvested and resuspended in fresh A^+^ medium supplemented with 10 mM sodium bicarbonate to a final OD_730_ = 1. Cell suspensions were maintained with continuous stirring and light. For dark respiration rates, cells were added to an oxygen-saturated medium kept in dark and the oxygen consumed was measured. The dissolved oxygen in the medium was measured using the DO probe.

### 4.4. Estimation of Biomass Composition

The total carbohydrate content of the biomass was estimated using the phenol sulfuric acid method [49]. Five mg dried cells were reconstituted in 1 mL of autoclaved Milli-Q water and dilutions were prepared. Five mL of ice-cold concentrated sulphuric acid was then added and the suspension was mixed by inverting three times and incubated at room temperature for 10 min. Fifty μL of saturated phenol was then added and the samples were incubated at 35 °C for 20 min. Finally, the absorbance was measured at 490 nm against a blank containing all the reagents except the biomass. The biomass samples were diluted depending on the content under varying conditions. The amount of carbohydrate was calculated using a standard plot of absorbance (A_490_) versus various glucose concentrations estimated using the same method.

For glycogen estimation, 1 mL of precooled methanol was added to 5 mg dried cells and vortexed [50]. The suspension was incubated at 60 °C for 15 min, cooled at room temperature and centrifuged at 8000 *g* for 10 min. The pellet was then washed with 100% ethanol. 100 µL of 40% KOH was added to the pellet, vortexed and incubated at 95 °C for an hour. Then, 200 µL of 100% ethanol was added to the solution after cooling and kept at −20 °C overnight to precipitate glycogen. The samples were centrifuged for 1 h at 13,000 *g*, supernatant was removed, and 40 µL of 2 N HCl was added to the pellet and incubation at 95 °C for 30 min. The sample was cooled to room temperature, and 40 µL of 2 N NaOH, 20 µL of 1 M phosphate buffer (pH = 7.0) and 40 µL of autoclaved Milli-Q water was added. The sample was vortexed thoroughly and glucose was then analysed via HPLC.

The total lipid was extracted using the Bligh and Dryer method [51] and measured gravimetrically.

Total protein was extracted from 10 mg dried cells using 1 mL of 1N NaOH [52]. The suspension was heated at 95 °C for 5 min and centrifuged at 4000 *g* for 15 min. The supernatant was collected and diluted as required. The protein concentration in the supernatant was measured using the Bicinchoninic acid method [53] (Pierce BCA Protein Assay Kit, Thermo Scientific, Rockford, IL, USA), using bovine serum albumin (BSA) as standard.

DNA was quantified using the Hoechst 33258 dye [54]. One mL of rehydration buffer (0.5 g lysozyme in 50 mL Tris-EDTA buffer of pH = 8.0) was added to 10 mg dried cells and incubated at 37 °C for 1 h. The dye stock solution (10 mg/mL) in TNE buffer (50 mM Tris-HCl (ph−7.4), 100 mM NaCl, 0.1 mM EDTA) was diluted to 2 µg/mL working concentration. Two hundred µL of the diluted dye was added to a 10 µl sample in a black plate and the fluorescence was measured using excitation at 360 nm and the emission at 460 nm. A commercially available DNA (Salmon sperm DNA, Sigma-Aldrich, St. Louis, MO, USA) was processed similarly as the samples and used as a standard.

RNA was isolated using perchloric acid method [55]. One mL of 0.3 M KOH was added to each tube having 10 mg dried cells and incubated at 37 °C for 60 min while mixing every 15 min. After 60 min of incubation, samples were cooled down to room temp. One mL of 3 M HClO_4_ solution was added to each tube and mixed properly. Samples were centrifuged at 8000 *g* for 10 min at 4 °C. The supernatant was transferred to a new labelled glass tube. The precipitate was resuspended in cold 0.5 M HClO_4_ and centrifuged again. The supernatant from this step was combined with the supernatant from the previous step. 0.5 M HClO_4_ was added to the pooled supernatant to make total volume up to 15 mL. The supernatant was then diluted two times with 0.5M HClO_4_ and quantified spectrophotometrically by taking absorbance measurements at 260 nm and 280 nm as per the standard formula [46].

Chlorophyll *a* and total carotenoids were extracted as per [56] and estimated spectrophotometrically. One mL methanol (precooled at 4 °C) was added to 1 mg dried cells. The suspension was mixed and covered with aluminium foil and incubated at 4 °C for 20 min. The sample was again centrifuged, and the absorbance of the supernatant was measured at the wavelengths of 470, 665 and 720 nm against methanol as blank. The concentrations of chlorophyll and carotenoids were then calculated as:

Chl *a* [μg/mL] = 12.9447 (A_665_ − A_720_), (1)

Carotenoids [μg/mL] = (1000*A_470_ − 44.76*A_666_)/221, (2)

Phycobiliproteins were extracted in phosphate buffer and cells were lysed by sonication. The concentrations were evaluated spectrophotometrically using absorbance values at 562, 615, and 652 nm for phycocyanin (PC), phycoerythrin (PE), and allophycocyanin APC), respectively [57].

The following equations were used to estimate the concentrations of PC, APC and PE in µg/mL:

[PC] = OD_615_ − 0.474(OD_652_)/5.34, (3)

[APC] = OD_652_ − 0.208(OD_615_)/5.09, (4)

[PE] = OD_562_ − 2.41(P C) − 0.849(APC)/9.62, (5)

The relative proportions of the constituents of soluble pool, inorganic ions and the content of the peptidoglycan were adopted from the *Synechocystis* model [19].

### 4.5. RNA Extraction, cDNA Synthesis and Transcriptional Analysis by RT-PCR

Total RNA was extracted using a commercial kit (Qiagen, Hilden, Germany). The cells were disrupted by crushing in pestle and motor in liquid nitrogen. The lysis and extraction steps were performed according to the manufacturer’s instructions. RNA quantity was estimated spectrophotometrically and the quality through gel electrophoresis. The RNA samples were treated with 1 U of RNase-free DNase (Thermo Scientific) according to manufacturer’s instructions. For cDNA synthesis, 2 µg of total RNA was transcribed with Revert Aid First Strand cDNA Synthesis kit (Thermo Scientific) in a final volume of 20 µL, following the manufacturer’s instructions. 20-fold standard dilutions of the cDNA were made and stored at −20 °C. The RT-qPCRs were performed on 96-well PCR plates covered with Optical Sealing Tape (Bio-Rad, Hercules, CA, USA). Reactions were manually assembled and contained 0.25 µM of each primer, 5 µL of iQ™ SYBR^®^ Green Supermix (Bio-Rad) and 20 ng of template cDNA in 10 µL reaction mixture. The PCR profile was: 3 min at 95 °C followed by 40 cycles of 30 s at 95 °C, 30 s at 62 °C and 30 s at 72 °C. Standard dilutions of the cDNA were used to check the relative efficiency and quality of primers. RT-qPCRs were performed with two biological replicates and technical triplicates of each cDNA sample in the iCycler iQ5 Real-Time PCR Detection System (Bio-Rad). The data obtained were analysed using the iQ5 Optical System Software v2.1 (Bio-Rad).

The transcripts levels of the glycogen synthesis genes: phosphoglucomutase (*pgmu1* and *pgmu2*), ADP-glucose-phosphorylase (*glgC*), glycogen synthase (*glgA1* and *glgA2*) were compared in *Synechococcus* sp. BDU 130192 and *Synechococcus* sp. PCC 7002 by RT-PCR. Negative controls (no template cDNA) were included and a melting curve analysis was performed in all assays. Efficiency values were calculated and the Cq values (cycle quantification value, that is the cycle number at which sample reaction curve intersects a threshold line) for each data set were exported to a Microsoft Office Excel file, the relative quantities of each sample were calculated using the gene-specific efficiency acquired from the dilutions series and normalized to the mean Cq value. Phosphoenolpyruvate carboxylase, a central enzyme in carbon concentrating mechanism, was used as the reference gene [58]. The primers used are given in Supplementary File (S5).

### 4.6. Phylogenetic Analysis

We reconstructed a phylogenetic tree based on the BLAST search of the 16S rRNA sequence of *Synechococcus* sp. BDU 130192 with the NCBI database [59]. The phylogenetic tree was reconstructed using the NCBI’s “Fast minimum evolution” algorithm. The tree was exported in “newick” format and reloaded in MEGA version 5.0 [60].

### 4.7. Reconstruction of the Genome-Scale Metabolic Model

**Figure 7:**
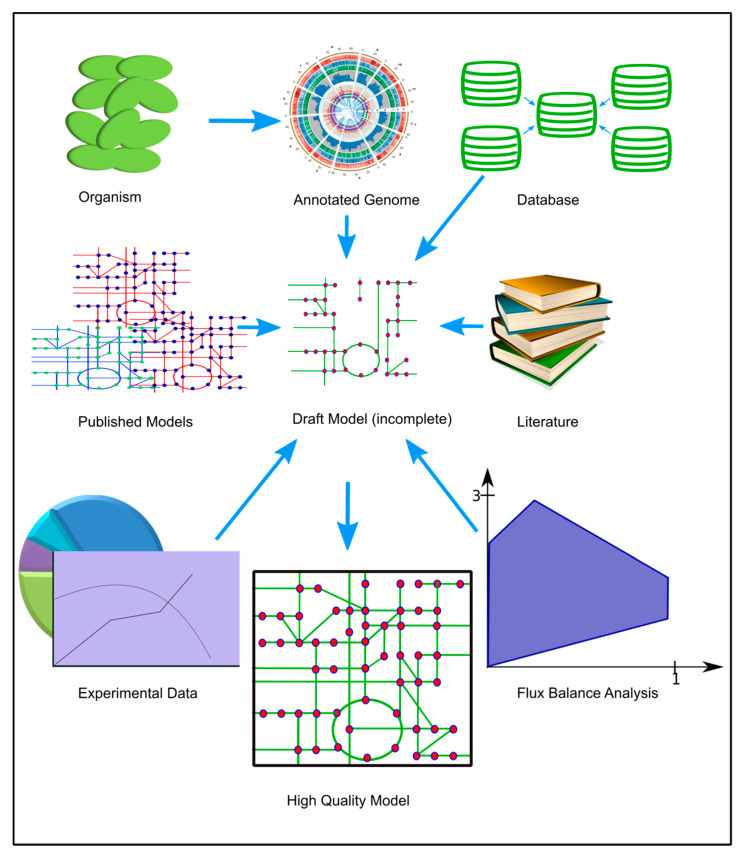
Diagram representing the steps to reconstruct metabolic models from its annotated genome. The genome of the organism was sequenced and annotated. A draft GSMM is obtained from the annotated genome. The draft model was refined iteratively using literature, biological database(s) and previously published models until a final curated model is obtained.

The genome sequence and the annotation information was taken from [11]. The genome scale metabolic model, *i*Syn706, was reconstructed from the annotated genome of this organism according to an established protocol [26,61]. The reconstruction of a genome scale metabolic model is an iterative process that starts with a draft model based on the annotated genome, which is gap-filled and refined until a complete model is obtained that reasonably describes the cellular metabolic response under different conditions. The biomass composition, which provides quantitative amounts of metabolites needed to make a gram of biomass, was measured (see method Section 4.4) and was used to construct the biomass equation. We have also taken information from previously published models [19,34,35], KEGG [62], BRENDA [63], BIOCYC [64] and METACYC [64] databases, wherever required as shown in Figure 7. The details are provided below.

#### 4.7.1. Draft Model

First, using the annotated genome which contained the information of genes and the Enzyme Commission (E.C.) numbers of metabolic genes, we prepared a list of all the metabolic enzymes present in the genome of the native cyanobacteria. Then, the draft model was reconstructed by extracting the reactions of all of the E.C. numbers and metabolic enzymes from KEGG [62]. The draft model was then refined iteratively as explained below until we obtained a mass-balanced GSMM that gave physiologically-relevant simulations. The exchange reactions enable the consumption or secretion of metabolites that can be consumed or secreted by the organism. These reactions were added to the model based on literature or genetic evidences. The added transport reactions transport the metabolite from one compartment to another compartment either by diffusion or by active transport. We have included the common transport reactions and based on annotation. Since cyanobacteria are prokaryotic, so they don’t have segregated compartments like eukaryotes. They have membrane-like organelles like thylakoids and periplasm. We have kept the well-established reactions in thylakoid and periplasm [19]. All the remaining reactions were kept in cytosol.

#### 4.7.2. Formation of the Biomass Equation, Biomass Formula and Biomass Degree of Reduction

The biomass equation was derived based on experimental measurements of the biomass composition. The stoichiometric coefficients in the biomass equation are the molar amounts of the individual components in 1 g of biomass. The amounts of total carbohydrates, glycogen content, lipids, proteins, DNA, RNA and pigments were normalized to the biomass (mg) to obtain the respective content in µmol/mg or mmol/g of dry cell weight. The measured biomass composition was used to generate the biomass equation as given in Supplementary File S6. The coefficients of biomass precursors in the biomass equation are the mmol of the precursor needed to form 1g of biomass. Biomass formula and its degree of reduction were calculated using the template provided in [36,45].

#### 4.7.3. Gap Filling and Model Refining

The gaps in the draft model were manually identified by performing optimisations for production of every biomass precursor (amino acids, carbohydrates, lipids, nucleotides etc.) one by one. Gap(s) in the pathways was/were identified if the model was unable to synthesize a required biomass precursor. We then searched the previously-published cyanobacterial genome-scale metabolic models [19,34] as well as KEGG database to identify the gap-filling reactions. In order to identify the presence of the genes encoding the gap-reaction(s), the genes encoding the gap reactions were taken from the closely-related cyanobacterial models and BLAST searches were performed against the genome sequence of *Synechococcus* sp. BDU 130192. The genes having an E-value of ≤ 10^−15^ were included in the model along with their associated reactions. However, some reactions were included in the model without any significant similarities to complete the pathway for biosynthesis of biomass precursors.

The thermodynamic infeasible loops are identified by fixing the ATP-Synthase reaction flux to some positive value (e.g., 1) while fixing all the exchange reactions to zero during optimization. If the optimization is still feasible then the reactions with non-zero fluxes are the infeasible loop reactions.

These loops were analyzed on a case-by-case basis and rectified by correcting the directionality of the reactions based on KEGG, BioCyc, MetaCyc databases as well as previously-published GSMMs.

Finally, the model was thoroughly checked for the presence of any thermodynamically infeasible cycles by testing for the production of ATP without any carbon source. Such infeasible cycles, if detected, were removed by correcting the directionality of some reactions in the cycle.

#### 4.7.4. Energy Requirements

Microorganisms require two types of energies: (a) Growth-associated ATP maintenance (GAM), which accounts for the energy required in synthesis of precursors, polymerisation to form DNA and proteins and (b) Non growth-associated ATP maintenance (NGAM), which accounts for the energy required to maintain cellular structure and integrity. The GAM energy contribution is taken into account as a coefficient of ATP in the biomass equation while the NGAM contribution was accounted by adding a separate ATP hydrolysis reaction and giving it a flux equal to the NGAM. As the values of GAM and NGAM are not available for this strain, these values were adapted from that of *Synechococcus* sp. PCC 7002 models, [34] for the GAM and [12] for the NGAM value respectively. Similar assumptions are regularly employed in cases where the actual values are not available, for example, in these previous studies [65,66].

### 4.8. Model Simulations for Autotrophic Condition and Reaction Essentiality Analysis

Flux balance analysis (FBA), a constraint-based approach that is used to calculate the internal fluxes distributions of metabolic models, was performed using the Cobra Toolbox [37] in MATLAB 8.4 (R2014b) and used the glpk (GNU Linear Programming Kit) solver. We used the minimization of total fluxes as the objective function (parsimonious FBA) for the simulations after constraining the biomass synthesis flux and oxygen-production flux range as this provides a reasonable flux distribution and has been employed in FBA of photoautotrophs [67]. For performing the FBA simulations, a linear programming problem was formulated as follows for minimization of the total fluxes:

(6) $$\begin{array}{l} \mathrm{Minimize}\;\overset{n}{\underset{i}{\sum }}v\\ \mathrm{subject}\;\mathrm{to}\;N.v=0\\ v{\;}_{\mathrm{Biomass}}=x\\ v{\;}_{\mathrm{oxygen}}{\;}_{\mathrm{Exchange}}=y\\ v{\;}_{\mathrm{ATP}}{\;}_{\mathrm{Maintenance}}=\mathrm{ATP}\mathrm{Maintenance} \end{array}$$

where, N is the stoichiometry matrix, v is the reactions flux vector, x is the experimentally observed growth rate and y is the measured oxygen evolution rate.

During the photoautotrophic simulations, CO_2_, photon and a few ions exchange fluxes were left free while the other carbon sources (such as glycerol, glucose, etc.) were constrained to carry zero flux. The metabolic reactions essential for growth in photoautotrophic condition were identified by fixing the flux of each reaction at a time to zero and maximizing growth. The deletions which resulted in biomass flux values of ≤ 10^−4^ h^−1^ were defined as essential reactions. The active reactions are those reactions whose flux values are ≥ 10^−8^ mmol∙(gDCW∙h)^−1^.

When comparing the flux distribution of *Synechococcus* sp. BDU 130192 with *Synechococcus* sp. PCC 7002, a GSMM of *Synechococcus* sp. PCC 7002 (*i*Syp708) was used to simulate the flux distribution in *Synechococcus* sp. PCC 7002 without any modifications, while our model (*i*Syn706) was used to simulate the flux distribution in *Synechococcus* sp. BDU 130192. The growth rates of the two strains were set to those measured experimentally.

### 4.9. Production of Industrially-Relevant Bio-Products

The model was utilized to explore the capabilities of the organism to produce some native and non-native (heterologous) industrially-relevant compounds. For each of the products considered (e.g., acetate, citrate, or succinate), the corresponding exchange reaction was set as the objective function to maximize its production while constraining the flux through the biomass reaction to be at least 80% of the wild-type photoautotrophic growth flux. Transport and exchange reactions were added to the model wherever required. For simulating synthesis of non-native products, the least number of reactions required for their synthesis were identified manually using the KEGG [62] database and added to the model as required. The maximum theoretical yields were calculated as the ratio of product flux divided by the carbon dioxide intake flux. The linear programming formulation to perform FBA for the production of industrially relevant products is as follows:

(7) $$\begin{array}{l} \mathrm{Max}\;c^{T}v\\ \mathrm{subject}\;\mathrm{to}\;(i)\;N.v=0\\ \;\;(\mathrm{ii})-1000\;\leq v\;\leq \;1000, \end{array}$$

(8) $$\begin{array}{l} \;Max\;yield\;\left(x\right)=\frac{vobj\;}{vc}, \end{array}$$

where, N is the stoichiometry matrix, *v* is the reactions flux vector and c is the objective function. In this case, the objective function is exchange reactions for product formation. *vobj* and *vc* are the fluxes through the objective function and carbon source, respectively.

### 4.10. Statistical Analysis

Experimental data are presented as mean ± standard deviation (SD) of three biological triplicates. Student’s *t*-test was conducted to identify statistical significance.

## 5. Conclusions

Our results show that *Synechococcus* sp. BDU 130192 is an attractive candidate for feedstock applications and also for photoautotrophic production of biochemical. The high quality manually curated genome-scale metabolic model of this cyanobacterium yielded information on the metabolic response of this organism and will provide a useful basis for further investigation of its metabolism and to design metabolic engineering strategies.

## Figures and Tables

**Table 1:** Biomass compositions of *Synechococcus* sp. BDU 130192 and *Synechococcus* sp. PCC 7002.

S. No.	Components	BDU 130192 (mg/mg DCW)	PCC 7002 (mg/mg DCW)
**1.**	Protein	0.41 ± 0.010 ^#^	0.61 ± 0.0062
**2.**	Total Carbohydrates Glycogen	0.52 ± 0.065 * 0.417 ± 0.036 *	0.25 ± 0.013 0.180 ± 0.007
**3.**	Total Lipids	0.0370 ± 0.0005 ^#^	0.046 ± 0.0017
**4.**	RNA	0.0441 ± 0.0016 ^#^	0.069 ± 0.0025
**5.**	DNA	0.0045 ± 0.00023 *	0.0021 ± 0.00037
**6.**	Chlorophyll	0.0049 ± 0.0006 ^#^	0.0154 ± 0.0012
**7.**	Carotenoids	0.0035 ± 0.0002	0.0039 ± 0.0004
**8.**	Phycobiliproteins	0.00031 ± 0.00004 ^#^	0.007 ± 0.0003

(* shows higher levels in carbohydrates and DNA, **^#^** shows lower levels of protein, lipids, RNA, chlorophyll and phycobiliproteins, *p* < 0.05).

**Table 2:** Comparison of the *i*Syn706 model with other published models of cyanobacteria.

Model Name	Species Name	No. of Genes	No. of Reaction	No. of Metabolites	No. of Active Reactions	No. of Essential Reactions	Reference
***i*Syn706**	*Synechococcus* sp. BDU 130192	706	908	900	502	450	This Study
***i*Syp708**	*Synechococcus* sp. PCC 7002	705	647	622	322	277	[35]
***i*Syp611**	*Synechococcus* sp. PCC 7002	611	589	579	344	297	[40]
***i*JN*678***	*Synechocystis* sp. PCC 6803	678	864	795	529	481	[19]
***i*JB*785***	*Synechococcus elongatus* PCC 7942	785	850	786	-	-	[21]
***i*Syn811**	*Synechocystis* sp. PCC 6803	811	956	911	-	-	[41]

## Supplementary Materials

The following are available online at https://www.mdpi.com/2218-1989/10/5/177/s1, S1: GSMM in sbml format, S2: All model reactions and metabolites, S3: Model in Matlab “mat” format, S4: List of Gap Filling Reactions, List of Active reactions, List of Essential Reactions, List of non-native reactions product analysis, S5: List of Primers for RT-PCR, S6: Calculation of biomass equation coefficients and the degree of reduction of biomass, S7: Description of the genes present in the genome of *Synechococcus* sp. BDU 130192, Comparison of our model to the model of *Synechococcus* sp. PCC 7002 and Photoautotrophic production of industrially-relevant compounds.
