# Supplementary material for: Biochemical Characteristics and a Genome-Scale Metabolic Model of an Indian Euryhaline Cyanobacterium with High Polyglucan Content
Source: Metabolites. 2020 Apr 29;10(5):177. doi: 10.3390/metabo10050177 (PMC7281201; doi:10.3390/metabo10050177)
Supplement: Supplementary file 1 [file metabolites-10-00177-s001.zip › SuppleFiles.v2/S5_List_of_Primers_Used_for_RT_PCR.pdf]

Primers list for RT-PCR

Gene name	Primer name	Sequence
ppc	130192_ref_F	AACTCGTCGAACGGCTCAATC
	130192_ref_R	TCTGTGTGGACGCTGTTACC
***pgmu*1**	130192_pgmu1_F	ATCGCTTTGGGGTTGTGGATG
	130192_pgmu1_R	GGATTCTTCCCCGCCAATGAG
***pgmu*2**	130192_pgmu2_F	TTTAGAGGCAATTCAGGTGGG
	130192_pgmu2_R	AGAGCCGCTGACGGTTTTTAC
glgC	130192_glgC_F	TATTCCTGATGCGGCCAAGG
	130192_glgC_R	CTAAAGGGTGGGATCGGCTG
glgA1	130192_glgA1_F	ACGGCAGTTAGGACATGACG
	130192_glgA1_R	GTGGGCAATTTGGGGTTTCC
glgA2	130192_glgA2_F	CGATCCACTGCACCGTTTTC
	130192_glgA2_R	AAGCGCATGTGGTCGTCTAA
