# Supplementary material for: Biochemical Characteristics and a Genome-Scale Metabolic Model of an Indian Euryhaline Cyanobacterium with High Polyglucan Content
Source: Metabolites. 2020 Apr 29;10(5):177. doi: 10.3390/metabo10050177 (PMC7281201; doi:10.3390/metabo10050177)
Supplement: Supplementary file 1 [file metabolites-10-00177-s001.zip › SuppleFiles.v2/S7_Supplimentary_Text.pdf]

1. Genes present in Synechococcus sp. BDU 130192 genome

Analysis of the annotated genome [11] revealed that the amino acid and protein metabolism subsystems have the highest number of genes (13% each), followed by the cofactor and vitamins subsystems (12%) and the "stress metabolism" (5%). Other subsystems like membrane transport, DNA metabolism, cell division and cell cycle have relatively lower number of genes compared to *Synechococcus* sp. PCC 7002. Figure 5 shows the distribution of genes among different subsystems. We found genes involved in exopolysaccharide synthesis in the genome. Genes encoding CRISPR-associated helicases Cas1, Cas2 as well as Cas4 were also found. The transporters for branched chain amino acids and cyanate were also seen. The cytochorome-*b6* complex has 7 genes while cytochrome c also has 7 genes whereas *Synechococcus* sp. PCC 7002 has 4 genes for cytochrome *b*6 complex and 5 for cytochrome *c* oxidase. The circadian clock proteins KaiA, KaiC as well as the circadian oscillation regulator KaiB are present in the genome.

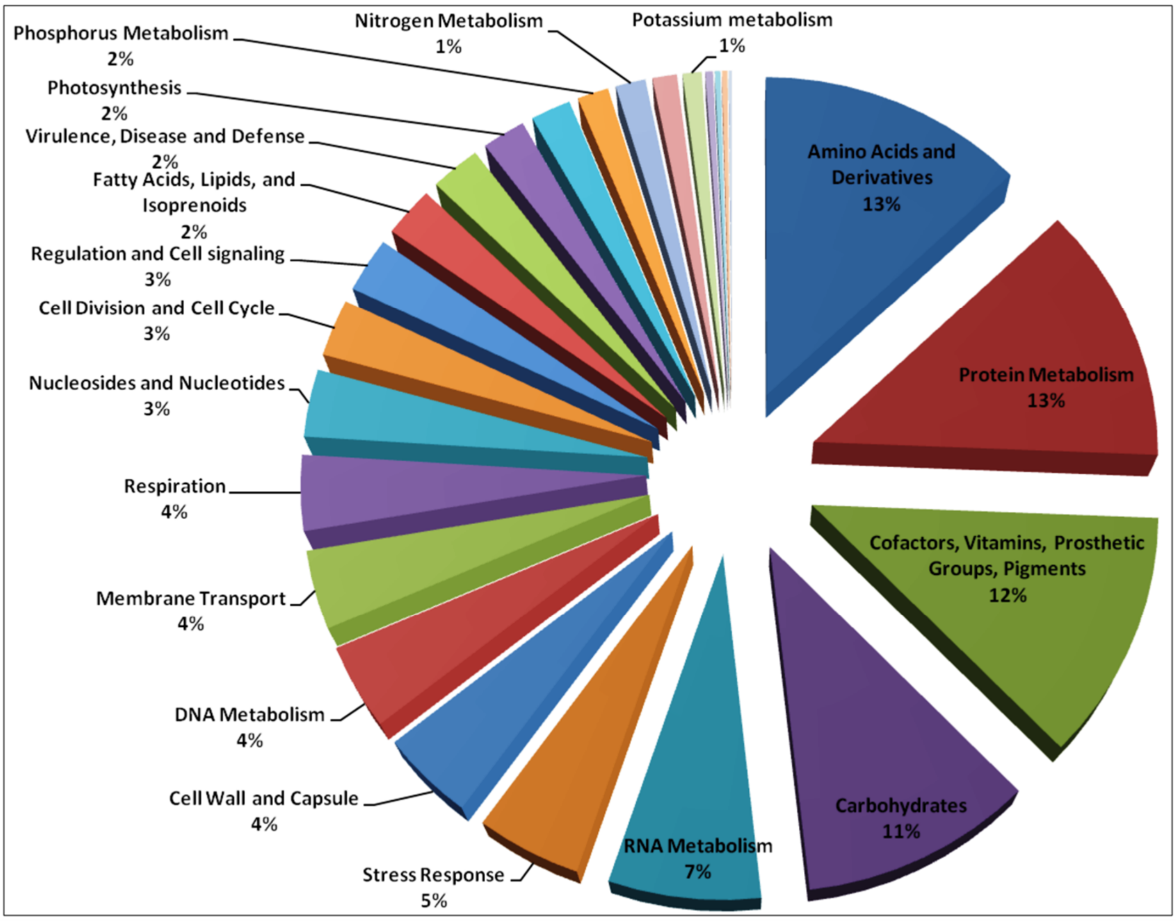

Figure S1. A pie-chart showing distribution of annotated genes among different subsystems.

2. Comparison of our model to the model of Synechococcus sp. PCC 7002

The model *i*Syn706 is the second-largest in terms of the number of reactions compared to other publically-available models of cyanobacteria (Table 2(a)).

We performed a pathway-wise comparison of *i*Syn706 model with the *i*Syp708 model. As given in Table 2b, *i*Syn706 has 908 reactions while *i*Syp708 has 647 reactions. The iSyn706 model contains 165 reactions while *i*Syp708 contains 115 belonging to amino acid metabolism. Further analysis revealed that *i*Syn706 has more reactions involving different amino acids compared to *i*Syp708. There are 31 and 6 reactions involved in phenylalanine, tyrosine and tryptophan metabolism in *i*Syn706 and *i*Syp708 models, respectively. Also, 27 and 12 reactions that are involved in 'cysteine and methionine

metabolism' are present in the *i*Syn706 and *i*Syp708 models, respectively. There are 10 and 3 reactions involved in histidine metabolism in *i*Syn706 and *i*Syp708 models, respectively. Only 4 reactions involved in folate metabolism are present in *i*Syp708 as against 15 in the *i*Syn706 model. In case of riboflavin metabolism, only 2 reactions are present in *i*Syp708 against 13 in the *i*Syn706 model. There are 81 and 60 reactions involving carbohydrate metabolism present in *i*Syn706 and *i*Syp708 models, respectively. Also, 85 and 54 reactions involving nucleotide metabolism are present in *i*Syn706 and *i*Syp708 models, respectively. So, the *i*Syn706 model has more alternative routes for synthesis of different biomass components. However, the reactions of alanine, aspartate and glutamate metabolism are slightly more in *i*Syp708 than in *i*Syn706, 28 vs. 24, respectively (Table 2(b)).

Table S1. Pathway-wise comparison between *i*Syn706 and *i*Syp708 models.

	***i*Syn706**	***i*Syp708**
**Phenylalanine, Tyrosine and Tryptophan Metabolism**	31	6
**Cystine and Methionine Metabolism**	27	12
**Histidine Metabolism**	10	3
**Alanine, Aspartate and Glutamate Metabolism**	24	28
**Carbohydrate Metabolism**	81	60
**Nucleotide Metabolism**	85	54
**Folate Metabolism**	15	4
**Riboflavin Metabolism**	13	2

3. *Photoautotrophic production of industrially-relevant compounds*

Table S2. Table showing the maximum theoretical yields of various native and non-native products under photoautotrophic conditions as per the *i*Syn706 model.

**S. No.**	**Product**	**Yield (mol/mol carbon)**	**No. of added Reactions**	**Added Genes**
	**Native**			
**1**	Acetate	0.09	0	
**2**	Succinate	0.05	0	
**3**	Pyruvate	0.06	0	
**4**	Citrate	0.03	0	
	**Non-Native**			
**5**	Acetone	0.06	3	atoB, {atoD or atoA}, adc
**6**	Phenyl Alcohol	0.02	2	ipdC, adhC
**7**	Butanol	0.05	6	Thl, Hbd, crt, bcd, adhE2 (adhE2 catalyzes 2 reactions)
**8**	Propane	0.05	6	Thl, Hbd, crt, bcd, adhE2, ADO
**9**	PHB	0.05	3	phaA, phaB, phaC
**10**	Ethanol	0.1	1	adhE

The growth rates were fixed at 80% of the wild type growth rate.

A homolog of alcohol dehydrogenase, which catalyzes the last reaction in ethanol formation, is present in the genome of *Synechococcus* sp. BDU 130192. However, no homolog for acetaldehyde

dehydrogenase was found. Therefore, ethanol synthesis requires only one additional (heterologous) reaction.
